# Rapidly Fatal Radiation-induced Glioblastoma

**DOI:** 10.7759/cureus.1336

**Published:** 2017-06-11

**Authors:** Ivy Ng, Char Loo Tan, Tseng Tsai Yeo, Balamurugan Vellayappan

**Affiliations:** 1 Radiation Oncology, National University Cancer Institute, National University Hospital Singapore; 2 Pathology, National University Hospital Singapore; 3 Neurosurgery, National University Hospital Singapore

**Keywords:** vestibular schwannoma, brainstem glioblastoma, secondary malignancy

## Abstract

Glioblastoma (GBM) typically occurs as a primary tumour (i.e., primary GBM) and predominantly affects elderly patients. The remaining ~10% occur as a result of malignant progression from lower grade astrocytic tumours (i.e., secondary GBM). Although there are no certain causative environmental agents, prior radiation exposure may play a role. We report on a patient who had been treated six years prior for a vestibular schwannoma with high-dose conventional radiotherapy and subsequently developed a rapidly fatal glioblastoma at the same location. The diagnosis was confirmed by routine histopathology as well as more advanced techniques, such as whole genome copy number analysis.

## Introduction

The use of radiotherapy in benign intracranial neoplasms is proven to be efficacious and is well-tolerated in the short-term. However, longer term risks, although remote, are present. These risks include radiation-induced malignancy, radionecrosis, and radiation-related neurocognitive decline. This should be carefully discussed with the patient, particularly if the patient is young. We present the diagnostic and management dilemma of a patient with a histology-proven vestibular schwannoma who was treated with conventional radiotherapy six years prior and subsequently developed a rapidly fatal brainstem glioblastoma (GBM) at the same location. 

## Case presentation

The patient had a history of vestibular schwannoma (VS) at the age of 37, six years prior. He had no family history or clinical signs of neurofibromatosis type 1 or 2. Due to the large size of the lesion, he was treated with a course of preoperative external-beam radiotherapy 66 Gray (Gy) in 33 fractions. This was followed by a planned left occipital craniotomy and tumour excision. Histology was consistent with a benign vestibular schwannoma. Postoperatively, he lost hearing function in the left ear but remained stable in clinical follow-up.

**Figure 1:**
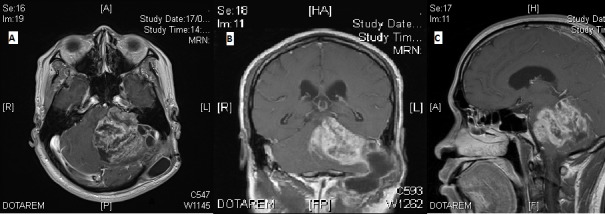
Magnetic resonance imaging at time of presentation A) Axial T1-weighted contrast-enhanced image; B) Coronal T1-weighted contrast-enhanced image; C) Sagittal T1-weighted contrast-enhanced image

He then complained of progressive unsteady gait and headache for several months. A magnetic resonance imaging (MRI) scan revealed a large enhancing extra-axial mass (5.5 x 6.1 x 5.2 cm) within the cerebellopontine angle (CPA) extending into the left internal auditory canal, in keeping with recurrent VS (Figure 1).

This lesion caused a mass effect upon the brainstem and cerebellum, and the associated oedema caused effacement of the fourth ventricle and mild tonsillar herniation. There was also a pseudomeningocele at the left cerebellopontine cistern. Clinically, he had nystagmus to the left and cranial nerve (CN) palsies on the left involving CN V, VII, VIII, IX, and X. There was reduced sensation to light touch and cold with pain sensation on the right side.

Based on radiological appearance, it was uncertain whether this could be a malignant transformation of the vestibular schwannoma. In view of the mass effect and aggressive radiological appearance, the patient underwent craniotomy and surgical resection. Intraoperative findings revealed a grayish vascular tumour adherent to the dura and pseudomeningocele. The tumour was maximally debulked up to the brainstem posteromedially.

**Figure 2:**
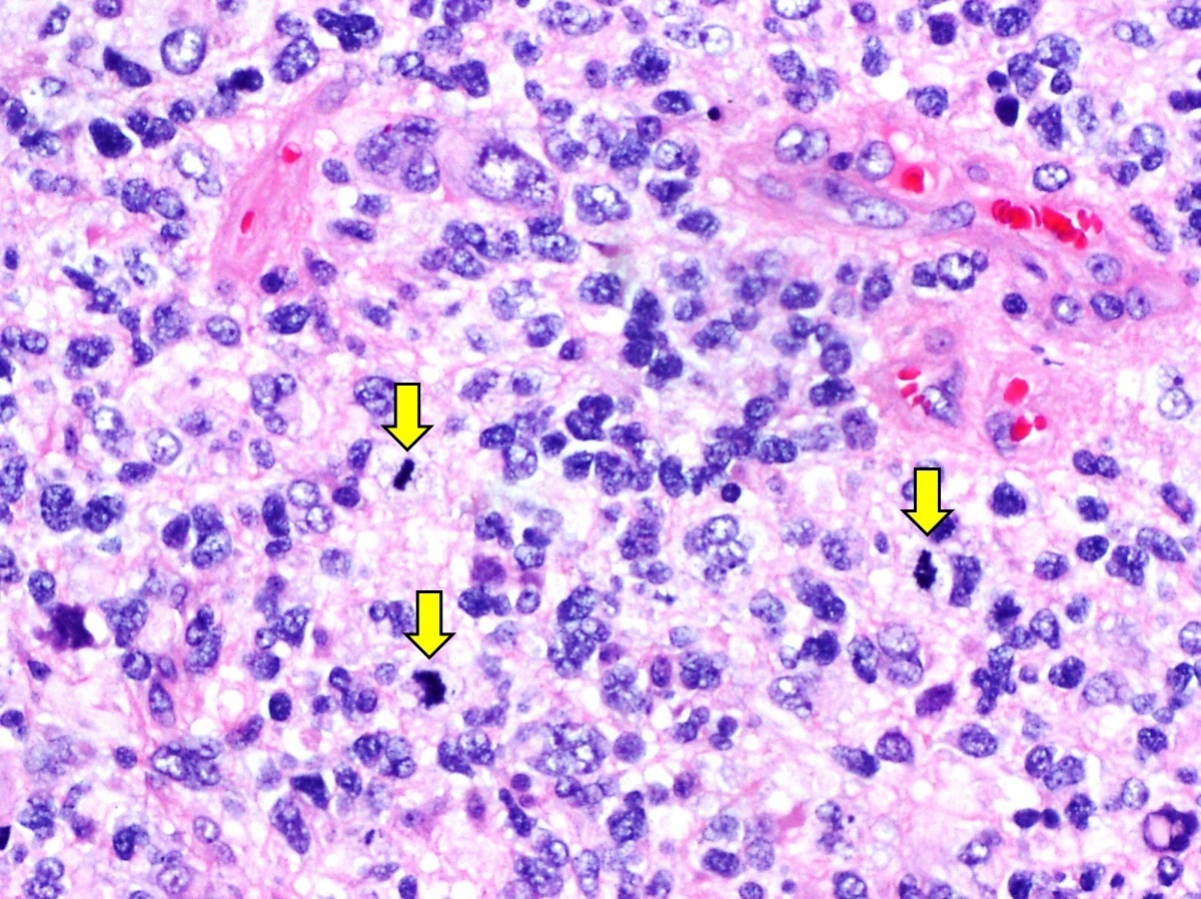
Histopathological examination of resected tumour Histological examination revealed a highly cellular tumour with marked nuclear atypia and numerous mitoses (yellow arrows) in fibrillary background. Necrosis and microvascular proliferation were also present (not shown).

Histology revealed a high-grade glial tumour composed of hypercellular sheets of anaplastic cells within a fibrillary background with numerous mitoses, areas of necrosis, and microvascular proliferation (Figure 2).

**Figure 3:**
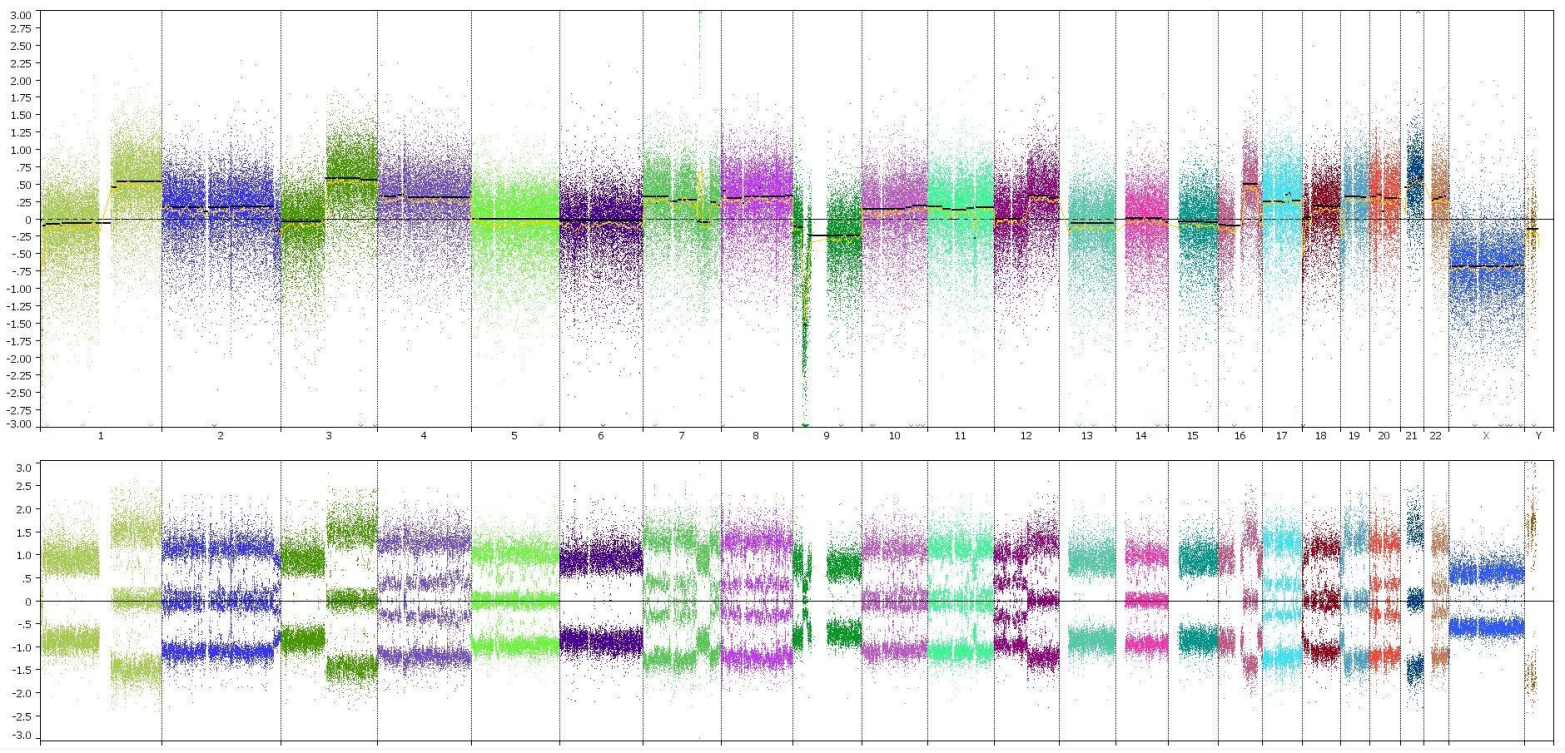
Whole genome copy number analysis Whole genome copy number analysis using OncoScan Affymetrix platform showed complex genetic aberrations, in particular, the absence of 1p19q whole arm codeletion and gain of chromosome 7p. There was also a gain in chromosome 10q.

The tumour cells were positive for glial fibrillary acidic protein (GFAP) and retained ATRX by immunohistochemistry. They were negative for isocitrate dehydrogenase 1 (IDH1 R132H) and the H3K27M antibody. Whole genome copy number analysis using the OncoScan® platform (Affymetrix, Santa Clara, CA, USA) showed the absence of 1p19q whole arm codeletion and mosaic gain of chromosome 7p (Figure 3).

No associated chromosome 10q loss was found. The features were those of a GBM. O^6^-methylguanine-DNA methyltransferase (MGMT) promoter methylation was not detected. 

In view of the histological findings, a spine MRI was performed to screen for drop metastasis. This revealed a small intrathecal enhancing nodule at the right side of the cauda equina at the level of L1. The differential diagnoses for this lesion were schwannoma or drop metastasis. As the patient was asymptomatic for this lesion, the clinical decision was made for close monitoring for progression.

**Figure 4:**
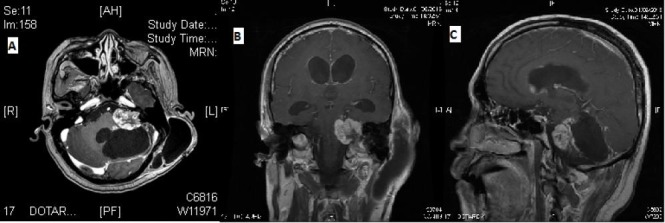
Magnetic resonance imaging performed post-surgery and re-irradiation A) Axial T1-weighted contrast-enhanced image; B) Coronal T1-weighted contrast-enhanced image; C) Sagittal T1-weighted contrast-enhanced image

The patient continued with poor functional status postoperatively, and hence, he was offered palliative re-irradiation alone. In view of the previous high dose received by the brainstem, a dose of 35 Gy in 10 fractions was delivered using highly conformal arc therapy. Repeat MRI brain done three months later showed the enhancing mass to be stable in size but with progression in the subependymal space and worsening hydrocephalus (Figure 4).

Consequently, the patient underwent an elective third ventriculostomy to relieve the pressure. Unfortunately, he continued to decline functionally and passed away four months after initial diagnosis.

## Discussion

This is a case of radiation-induced glioma (RIG) in a young patient, which fulfills Cahan’s criteria [1]. The second histologically distinct tumour arising from the original radiation field was also confirmed by genome copy number to be an aggressive glioma with poor prognostic features. Although this technique was helpful in confirming the diagnosis, there are no known markers to distinguish radiation-induced GBM from primary GBM. From the presentation of symptoms arising from the second tumour, this case was challenging in terms of diagnosis and treatment strategy.

Malignant transformation of a VS is exceedingly uncommon [2], and the risk is known to be higher in NF-2-associated VS [3]. The majority of the cases of malignant transformation appear to be sarcomatous [3]. A recent review has suggested that the risk of secondary malignancy after stereotactic radiosurgery to be 0.04% at 15 years [4]. In comparison, the risk after conventionally fractionated radiotherapy appears to be slightly higher as reported in a series of patients with pituitary adenoma who received radiotherapy. The cumulative risk of secondary brain tumours was 2.0% and 8.5% for 10-year and 30-year intervals, respectively [5]. Approximately half of these were meningiomas. In another series, the risk of RIG was estimated by Tsang, et al. to be 1.7% and 2.7% in the 10-year and 15-year intervals, respectively [6].

Limited studies have shown no distinguishing histologic and molecular characteristics of RIG from primary GBM [7]. Although we were unable to prove pathologically that this patient had RIG, other supporting features included his age of presentation and location of the tumour. The incidence of GBM peaks in the 75-84 age group [8] and is uncommon in the cerebellopontine angle, as seen in our patient.

Paulino, et al. have suggested that patients with RIG, who undergo re-irradiation, have a longer survival than the patients who do not [9]. This is understandable due to the dose-response seen with glioma [10]. However, many clinicians may be reluctant to offer re-irradiation to the full dose, given the potential cumulative neurotoxicity. Our patient was particularly challenging to manage as the brainstem had previously been irradiated to tolerance (54 Gy).

Advanced techniques, such as proton or heavy ion therapy, may be useful in re-treatment of similar cases. Future research in molecular profiling may uncover targetable mutations present in RIG, which would provide more therapeutic opportunities for this group of patients. 

## Conclusions

The management of RIG remains challenging. The risk of RIG, although low, should be carefully considered before patients undergo radiotherapy. This is particularly so for young patients with benign pathology who are expected to have a normal life expectancy. Furthur research in the molecular profiling of RIG and advanced re-irradiation techniques may help in the management of this group of patients. 
